# The Role of Suppressors of Cytokine Signalling in Human Neoplasms

**DOI:** 10.1155/2014/630797

**Published:** 2014-03-16

**Authors:** Walid Sasi, Anup K. Sharma, Kefah Mokbel

**Affiliations:** ^1^St George's Hospital Medical School, University of London, Cranmer Terrace, London SW17 0RE, UK; ^2^The London Breast Institute, The Princess Grace Hospital, 42-52 Nottingham Place, London W1U 5NY, UK

## Abstract

Suppressors of cytokine signalling 1–7 (SOCS1–7) and cytokine-inducible SH2-containing protein (CIS) are a group of intracellular proteins that are well known as JAK-STAT and several other signalling pathways negative feedback regulators. More recently several members have been identified as tumour suppressors and dysregulation of their biological roles in controlling cytokine and growth factor signalling may contribute to the development of many solid organ and haematological malignancies. This review explores their biological functions and their possible tumour suppressing role in human neoplasms.

## 1. Cytokines and Their Signalling Pathways

Cytokines are a large family of secreted soluble glycoproteins that regulate cellular growth and differentiation which are part of fundamental biological processes including embryonic development, immunity, wound healing, and haematopoiesis. Cytokines carry information about the biological status to target cells by interacting with receptors on the cell surface. Cellular responses to cytokine stimulation depend on the type of cytokine and the nature of the target cell and include proliferation, differentiation, effector function, and survival [1, 2]. Cytokines activate multiple intracellular signalling pathways in order to produce their physiological effects. One of the most studied pathways is that involving the receptor-associated janus kinases (JAKs) and the latent cytoplasmic transcription factors signal transducers and activators of transcription (STATs) [3, 4]. Genetic deletion experiments in mice have demonstrated that this pathway is critical for the actions of specific cytokines. For example, STAT1 is absolutely required for the actions of interferons, STAT4 is absolutely necessary for the actions of interleukin-12 (IL-12), STAT6 is required for the actions of interleukin-4 (IL-4), and JAK3 is required for the actions of cytokines that use the common *γ* receptor [5]. This cascade requires strict cellular control and loss of regulation can promote tumorigenesis and chronic inflammation. The threshold, magnitude, and specific responses elicited by cytokine stimulation are regulated by numerous mechanisms including tyrosine phosphatases, receptor internalisation, proteasomal degradation of signalling adaptor molecules, soluble receptor antagonists, and specific inhibitors, including the protein inhibitors of activated STATs (PIAS) and suppressor of cytokine signalling (SOCS) proteins.

The expression of SOCS proteins can be induced by cytokine stimulation, and they not only serve to interfere with signalling from the inducing cytokine in a classic “negative feedback” loop but also regulate signalling downstream of other cytokines, a process known as “cross-talk.”

## 2. The Mechanism of Action of the JAK-STAT Pathway

**Figure 1:**
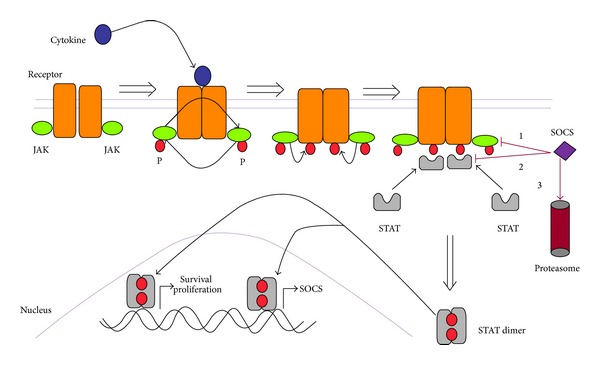
Activation and regulation of the JAK-STAT pathway. STAT homodimers or heterodimers translocate to the nucleus. Cytokines signal by approximating receptors and associated janus kinases (JAKs), initiating a cascade of phosphorylation (P). This results in the phosphorylation and dimerisation of STATs, which translocate to the nucleus initiating gene transcription. In addition to genes involved in survival, proliferation, and function, STATs initiate transcription of SOCS. SOCS inhibits cytokine signaling by (1) binding to JAKs and directly inhibiting their kinase activity; (2) blocking STAT recruitment to the cytokine receptor; and (3) targeting the receptor or its JAK for degradation by the proteasome.

Although cytokine receptors lack intrinsic kinase activity, they are constitutively associated with members of JAK family of protein tyrosine kinases, which include JAK1, JAK2, JAK3, and tyrosine kinase 2 (TYK2). The cytokine mediated phosphorylation requires activation of these receptor associated JAKs [6, 7]. These 4 JAKs can be activated by different cytokines and receptors (Table 1). The main steps in this pathway are shown in Figure 1.

## 3. Other Signalling Pathways Are Activated in a Similar Manner

In a similar way to STATs activation, cytokine stimulation initiates multiple signal transduction cascades such as those involving RAS, phosphatidylinositol 3-kinase (PI3 K), and phospholipase C-*γ*. Together, these pathways result in the regulation of gene expression in the nucleus, leading to target cell differentiation, proliferation, survival, apoptosis, or activation [1].

## 4. The STAT Family: Their Structure and Basic Function

The STAT family of transcription factors consists of STAT1, STAT2, STAT3, STAT4, STAT5a, STAT5b, and STAT6 [16–18].

**Figure 2:**
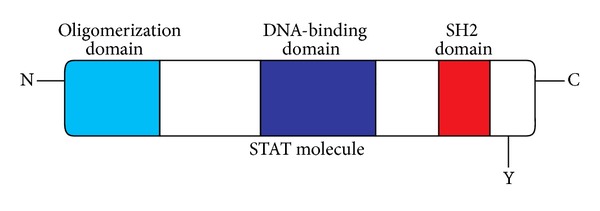
Structural characteristics of STAT proteins.

As shown in Figure 2, STAT proteins have a structural homology with a conserved carboxy-terminus SH2 domain, a central DNA-binding domain, and an amino-terminus oligomerization domain [4, 17–19]. STATs bind to activated cytokine receptors through their SH2 domain, resulting in JAK-induced phosphorylation of a conserved tyrosine residue (Y) on the C-terminus. Interactions between the phosphorylated tyrosine and SH2-domain result in the formation of STAT dimers. Phosphorylated STAT dimers translocate to the nucleus where they bind to DNA (via the DNA-binding domain) and activate target gene transcription. The helical N-terminus is highly conserved and is involved in the formation of STAT oligomers.

## 5. The Structure of SOCS Proteins

**Figure 3:**
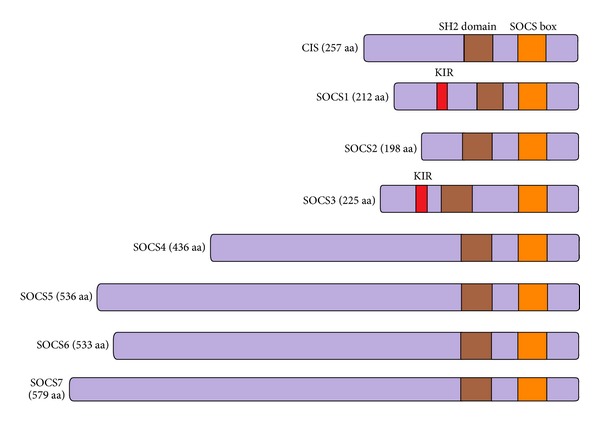
SOCS family members.

SOCS family of proteins comprises 8 proteins: SOCS1–7 and cytokine-inducible SH2-containing protein (CIS). They appear to be induced by cytokine stimulation to act as negative regulators of signalling in a classical negative feedback loop [20, 21].  Figure 3 shows the general structure of SOCS proteins. Interestingly, a comparison of the primary amino acid sequence and genomic structure of SOCS family members shows that pairs of SOCS proteins are more similar to each other than to other SOCS proteins. Indeed, CIS and SOCS2, SOCS1 and SOCS3, SOCS4 and SOCS5, and SOCS6 and SOCS7 all form related pairs [22]. SOCS2 and CIS exhibit approximately 35% amino acid identity [23, 24], whereas the rest of the family members are more distantly related and share approximately 25% of their sequences. Some of the human SOCS proteins have quite high homology with those found in mice and rats: for example, SOCS1 in both of these species shares 95–99% amino acid identity with human SOCS1 [23].

### 5.1. The N-Terminal Domain

Members of the SOCS family contain N-terminal regions of variable length (50–380 amino acids) and share little sequence similarities in that region. For example, CIS, SOCS1, SOCS2, and SOCS3 have relatively short (50–80 residues) N-terminal regions, whereas SOCS4, SOCS5, SOCS6 and SOCS7 have longer N-terminal regions of up to 380 residues [22, 25, 26]. Early studies showed that N-terminal regions of SOCS family members have no recognizable motifs, the exception being SOCS7 which contains a putative nuclear localization signal and multiple proline-rich regions [27]. More recent reports showed evidence that N-terminal domain contains an extended SH2 subdomain (ESS) that contributes to substrate interaction [28–30].

### 5.2. The SH2 Domain

All eight proteins were found to contain a central SH2 domain of approximately 95 amino acids [31–33]. It interacts in a context-specific manner with phosphotyrosine residues of the SOCS-target proteins, including cell surface receptors, resulting a characteristic target specificity of the SOCS members [34].

### 5.3. The C-Terminal Domain

Similarly, all eight proteins were found to share a conserved 40-residue C-terminal motif termed the* SOCS box* [22, 23]. This particular motif is important in ubiquitin-mediated proteasomal degradation by SOCS proteins. The SOCS box is comprised of two functional subdomains: a BC box that recruits Elongin B and C and a Cul box that mediates Cullin 5 binding. The resulting complex is able to bind RBX2, leading in turn to the recruitment of the remaining components of an E3 ubiquitin ligase complex [35, 36].

## 6. The Molecular Mechanism of Action of SOCS Proteins 

**Figure 4:**
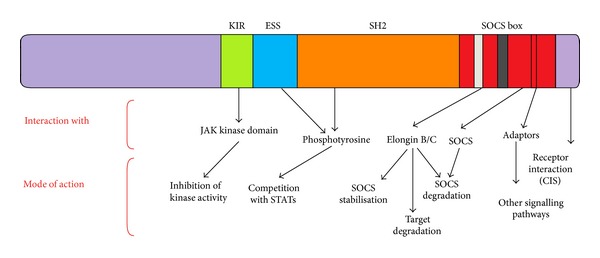
Versatility of SOCS protein functions. The different domains of SOCS proteins mediate distinct interactions and functions. Some of these are specific for certain SOCS family members such as the KIR-dependent inhibition of JAK activity by SOCS1 and SOCS3 and SOCS box-dependent substrate recognition in case of CIS. Other functions are more general such as competition for shared receptor motifs and Elongin B/C recruitment. The SOCS box is involved in as diverse as receptor interaction, adaptor coupling, target degradation, and control of SOCS protein stability.

SOCS proteins can modulate cytokine receptor signalling by multiple complementary mechanisms (Figure 4).  Table 2 shows the various associations of SOCS family members with many cytokines and growth factors.

### 6.1. SOCS1* In Vitro* Function

SOCS1 inhibits signalling by a wide range of cytokines including LIF, IL-6 [23, 24, 32], IL-4 [57], GH [47, 54, 60], PRL [42, 132], TPO [23], interferons [61, 133], and stem cell factor (KIT ligand) [133]. It interacts directly with the kinase domain (JH1) of JAKs (JAK1, JAK2, JAK3, and Tyk2) via its ESS and KIR domains and inhibits their kinase activation and catalytic activity as well as subsequent phosphorylation and activation of downstream substrates such as the STAT proteins [22, 24, 31, 32, 96]. JAK2 can be phosphorylated on several tyrosine residues, but phosphorylation of a single tyrosine residue at position 1007 (Y1007) appears to be an early and critical requirement for catalytic activation. This results in direct binding of SOCS1 to the activation loop of JAKs [28]. SOCS1 can also interact with Elongin B/C and Cullin 5 through its SOCS box, leading to ubiquitination and proteasomal degradation of target substrates [35, 134], such as JAK1 [134], JAK2 [135], TEL-JAK2 [136, 137], GEF, VAV [138], insulin receptor substrate (IRS)-1, and IRS-2 [139], as well the TLR2/4 adaptor protein MAL [75]. In fact, SOCS1 interaction with TLR adaptor proteins such as MAL and IRAK is now known to be one of two main mechanisms by which SOCS1 regulates TLR signalling [65, 75, 140]. The other mechanism is by interference of JAK-STAT signalling downstream of TLR. The SOCS box has also been shown to confer protection on SOCS1 against proteolytic degradation [141]. SOCS1 has also been shown to interact with other activated kinase domains such as those from TEC [142], KIT, FLT3, CSF-1 receptor (C-FMS), and PDGF receptor [69], likely through its SH2 domain. It also binds to the signalling molecules VAV, GRB2, P85, NcK, ITK, and FGH through interaction of their SH3 domains with proline motifs in the N-terminal domain of SOCS1 [69]. While the binding of SOCS1 to JAKs and TEC leads to inhibition of their kinase activity, this does not occur upon the binding of SOCS1 to activated KIT receptors despite that SOCS1 inhibits the proliferative stimulation of haematopoietic and fibroblast cells mediated by this receptor [69]. More recent studies have shown another interesting action of SOCS1 which involves interaction with phosphotyrosine residues on the IFN*α*R1 and IFN*γ*R1 receptor subunits in a JAK1-independent manner [143, 144]. Furthermore, through the JNK pathway, TNF*α* was found to induce miR-155 which in turn targets SOCS1 at its 3′UTR [145]. This interaction was further demonstrated by knocking down miR-155 in mouse osteoblastic cells which has resulted in increased SOCS1 protein expression following TNF*α* stimulation, while transfection with miR-155 has inhibited wild-type SOCS1 [145] and also was demonstrated in T cells where FOXP3 contributes to the maintenance of SOCS1 levels by negatively regulating miR-155 [146].

### 6.2. SOCS2 and CIS* In Vitro* Functions

SOCS2 and CIS form a second group of related proteins that are more ubiquitously expressed than SOCS1 and SOCS3 but are relatively poor inhibitors of the actions of most cytokines. Neither CIS nor SOCS2 can bind to the JAKs or inhibit JAK activity [28, 42]. SOCS2 controls signalling by GH as expression of SOCS2 in human embryonic kidney 293 cells and COS-1 cells were found to inhibit GH-mediated STAT5 signalling [47, 89]. SOCS2 also regulates signalling induced by other cytokines such as PRL [147], LIF [78], IL-2, IL-3 [79], and IL-6 [96], and also by growth factors, such as EGF [83] and IGF-1 [70]. SOCS2 function differs from other SOCS family members in two ways. Firstly, SOCS2 appears to play a dual regulatory role, both inhibiting and potentiating signalling depending on its concentration and cellular context [42, 89, 96].* In vitro* studies have demonstrated that low levels of SOCS2 led to a reduction in GH signalling, while higher levels of y* increased* GH signalling [89].

Secondly, SOCS2 has been shown to possess the ability to antagonise other SOCS family members [43]. Cotransfection studies have shown that SOCS2 was able to block the inhibitory effects of SOCS1 (but not that of SOCS3) on GH signalling in a SOCS2-dose dependent manner [89], and SOCS2 was shown to exhibit an antagonistic role in the SOCS1- and SOCS3-mediated negative regulation of IL-2 and IL-3 signalling, respectively [79]. SOCS2 is thought to primarily exert its effects by stimulating ubiquitination of target proteins, including receptors, such as GHR [148], and signaling proteins, such as SOCS3 [79]. Interestingly, SOCS2 was first cloned using a yeast two-hybrid system with the IGF-I receptor as a bait [70]. In contrast to many cytokine receptors that lack intrinsic kinase activity and interact with JAKs to initiate phosphorylation cascades, the IGF-I receptor is a member of the tyrosine kinase receptor family and autophosphorylation of the IGF-I receptor stimulates tyrosine kinase activity and is required for SOCS2 binding. The gigantic phenotype of the SOCS2-deficient mice and the results of the above studies suggest an important role for SOCS2 in the regulation of growth, possibly by modulating GH and IGF-I signalling [47, 70, 89, 149]. CIS can interact with phosphorylated tyrosines in the cytoplasmic domains of several cytokine receptors such as GHR, EPOR, TPOR, IL-3 R, and IL-2 R*β* [31, 39, 46–48, 54, 150], and SOCS2 can interact with the activated IGF-I receptor [70]. CIS inhibits GH-induced STAT5b activation, as both CIS and STAT5b bind to an overlapping set of phosphotyrosine residues on the GH receptor [46, 47, 54]. CIS is induced by STAT5 in response to EPO and IL-3 stimulation and binds EPO receptor and IL-3 receptor *β* chain in a phosphorylation-dependent manner [23, 39]. Overexpression of CIS inhibits EPO-dependent STAT5 activation and has been suggested to inhibit signalling by competing for the phosphorylated receptor residues that act as docking sites for STATs [39]. Indeed, CIS associates with phosphorylated Y401 of the EPO receptor, which is one of the two STAT5-binding sites in this receptor, while the other is at Y343 [150]. Furthermore, low levels of STAT5b expression potentiate the inhibitory action of CIS which suggests that CIS competes with STAT5b for activated GH receptor binding [37]. Thus, Matsumoto et al. [37] proposed that CIS could inhibit cytokine signalling by blocking access of STAT5 to tyrosine-phosphorylated receptors. EPO-induced STAT5 activation still occurs when Y401 on the EPO receptor is mutated to phenylalanine, but this could be because the binding of STAT5 to Y343 on the EPO receptor is sufficient for activation [151, 152]. Thus, the fact that CIS interrupts the binding of STAT5 to Y401 on the EPO receptor does not fully explain the mechanism by which CIS inhibits EPO signalling. CIS may act by a mechanism other than simple competition with STATS for receptor binding. Indeed, in a report by Li et al. [55], CIS was found to be an early response gene induced by T cell receptor (TCR) stimulation via an alternative pathway not involving STAT5. CIS can also negatively regulate signalling by mediating proteasomal degradation of activated receptor complexes via interactions between its SOCS box, Elongin B/C, and Cullin 5 [153, 154]. Recent studies suggest posttranscriptional regulation of CIS by micro-RNAs (miRs), such as miR-98 or let-7 that target the 3′ untranslated region (UTR) of the CIS mRNA, to cause translational repression. Bacterial LPS was able to decrease expression of miR-98 and let-7* in vitro*, thereby relieving the miR-mediated CIS translational suppression [155]. The CIS 3′UTR also contains ATAA destabilisation motifs, while the CIS protein possesses PEST sequences, which lead to rapid turnover of the mRNA and protein, respectively [31].

### 6.3. SOCS3* In Vitro* Functions

SOCS3 inhibits many of the same cytokine/growth factor receptor systems as SOCS1 (Table 2), including LIF/IL-6, IL-4, GH, IFN, and PRL. It has also been shown to inhibit signalling by IL-2 and IL-3 [85] and leptin [51, 102, 103, 156]. However, it appears to be a weaker inhibitor of IFN signalling than SOCS1 [61, 133]. At high levels, SOCS3 can interact with JAKs, although it has a lower affinity than that observed for SOCS1 and must be expressed at a significantly higher level than SOCS1 for equivalent inhibition of kinase activity [33, 42, 157]. The mechanism of action of SOCS3 is different from that of SOCS1. Although SOCS3 is demonstrating low affinity binding to JAK2, it does not appear to inhibit JAK1 or JAK2 kinase activity* in vitro* [33, 42, 96, 157]. Both SOCS1 and SOCS3 coimmunoprecipitate with JAK2, but only SOCS1 significantly inhibited the* in vitro* kinase activity of JAK2 [96]. However, SOCS3 can inhibit the activation of JAK2 in response to GH stimulation when both SOCS3 and the GH receptor are coexpressed in HEK 293 cells. Furthermore, in the GH receptor system, the interaction of SOCS3 with JAKs and its ability to inhibit kinase activity is enhanced by the presence of the activated receptor. This occurs, for example, when the expression level of GH receptor is increased, suggesting that optimal inhibition of JAK2 occurs when SOCS3 is bound to the GH receptor [54]. Similarly, SOCS3 both inhibits IL-2 signalling and associates with the activated IL-2R*β* chain, and its ability to inhibit JAK1 activity is significantly augmented in the presence of the IL-2R*β* chain which suggests that receptor association is necessary for maximal inhibition [85]. SOCS3 also binds to the leptin and EPO receptors, and mutation of the SOCS3 binding site on these receptors interferes with the ability of SOCS3 to inhibit leptin and EPO signalling, respectively [93, 158]. The SOCS3-SH2 domain was also initially shown to interact with Y1007 in JAK2, albeit with slightly lower affinity [158], but subsequent studies demonstrated a high affinity interaction with other phosphotyrosine residues located within receptor subunits, namely, Y757 and Y759 In fact, by comparing the binding affinity of SOCS3 for phosphopeptides derived from JAKs, STATs, and the gp130 subunit of the LIF/IL-6 receptor systems, it appears that the highest affinity was for peptides centred on the SHP2 binding site of gp130 around Y757 and Y759 (Table 3). In agreement with this is the finding that mutations of Y757 to phenylalanine on gp130 significantly reduced the capacity of SOCS3, but not SOCS1, to inhibit LIF/IL-6 signalling [113, 114]. Additionally, SOCS3 extended SH2 domain binds to the tyrosine phosphorylated receptor Y759, leading to the assumption that the inhibitory effect of SOCS3 depended on the interaction of the extended SH2 domain with pY759 in the receptor protein. SOCS3, therefore, in contrast to SOCS1, has to be recruited to the receptor complex in order to inhibit IL-6 signal transduction [114]. SOCS3 may also inhibit the kinase activity of JAKs through its pseudosubstrate region, KIR, in the same way as SOCS1, but only after recruitment and binding to a critical phospho-tyrosine at the intracellular part of the cytokine receptor, Y757 or Y759, in the case of gp130 [158].

The above observations that SOCS3 preferentially and competitively binds to the binding site of SH2-domain haematopoietic phosphatase (SHP2) on the gp130 and others regarding SOCS3 interaction with leptin receptor [112, 113] support the idea that the N-terminal domains of SOCS1 and SOCS3 are indeed functioning in a similar manner and that SOCS3 can act to inhibit JAK activation, but only when recruited to the appropriate site on an activated receptor [115]. More definitive evidence has shown that KIR domain in SOCS3 is necessary for its JAK inhibitory functions and that point mutations in this region have abrogated this inhibition [159].

Now, as SHP2 has been shown to mediate positive signalling by IL-6-type cytokines by activating the RAS-MAPK pathway, the competition of SOCS3 binding with SHP2 on gp130 suggests that it may also inhibit this pathway as well as the JAK-STAT pathway, compensating for its relatively poor affinity for the JAKs. It follows that much of the evidence cited to suggest that SHP2 can also inhibit gp130 signalling pathways becomes difficult to interpret and needs to be reviewed because many of the experimental systems used (such as receptor mutations or dominant negative SHP2) could now be explained by inhibition of SOCS3 binding to this same receptor site [160]. Similarly, the recently described phenotype of mice with phenylalanine mutation of Y757 in both alleles of the gp130 gene (splenomegaly, lymphadenopathy, enhanced acute phase responses, and hyperimmunoglobulinemia) could reflect the effect of loss of function of SHP2, SOCS3, or both [161].

Taken together, these studies suggest that SOCS3 action shares elements of that of CIS and SOCS1 and exerts its inhibitory action through 2 steps; first; it is recruited into the vicinity of the JAKs (but not JAK itself) by binding to activated cytokine receptors, and second, once localized at the receptor, SOCS3 likely inhibits JAK activity through its KIR. Evidence also supports other roles for SOCS3, including competition on receptor binding sites with substrates such as SHP2 [113] and STAT4 [162]. Finally, and like other SOCS members, it may target substrates for degradation [96].

### 6.4. SOCS 4–7 Proteins* In Vitro* Functions

Considerably less work has been done on the remaining two pairs of SOCS proteins, SOCS4 and 5 and SOCS6 and 7. In general, SOCS proteins such as CIS and SOCS2 can function by blocking access to phosphotyrosine residues and targeting proteins for ubiquitination and proteasomal degradation. Although it is tempting to extrapolate this well-defined function to other members of the SOCS family, it is evident that a unique mode of receptor recruitment may be involved [27, 119, 121, 122]. Most striking are the extended N-terminal regions of SOCS4 (270 aa), SOCS5 (368 aa), SOCS6 (369 aa), and SOCS7 (385 aa) (excluding the ESS), suggesting these four proteins form a subgroup within the SOCS family. Some authors have adopted a view based on recent research evidence suggesting that while CIS and SOCS1–3 are most often associated with regulation of cytokine receptor signalling through the JAK-STAT pathway, SOCS4–7 predominantly regulate growth factor receptor signalling via the control of receptor tyrosine kinases (RTKs) by target protein degradation and in the case of SOCS4 and SOCS5 also binding site competition [121, 129], while SOCS7 has been shown to directly bind signaling proteins to prevent their nuclear translocation and inhibiting their signal transmission [128]. However, the distinction of SOCS functions into cytokine receptor and RTKs is not strict.

#### 6.4.1. *In Vitro* Functions of SOCS4 and SOCS5

SOCS4 and SOCS5 share greater sequence similarity with each other than with other members of the SOCS family [22], with conservation largely restricted to the SH2 domain (92% amino acid identity) suggesting that while the SH2 domains may have an overlapping binding specificity [22], the N-terminal regions will have unique protein targets. Some* in vitro* studies suggested that SOCS4 and SOCS5 might regulate EGF signalling [119, 121]. Two ways of interaction with the EGF receptor have been identified: a phosphorylation-dependent interaction via the SOCS4-SH2 domain and Y1092 in the EGF receptor cytoplasmic domain and a phosphorylation-independent interaction via the SOCS5 N-terminal region [29, 119, 121]. Docking of SOCS4 to phosphotyrosine residues on the activated EGFR may subsequently result in targeting the receptor for proteasomal degradation by recruitment of E3 ubiquitin ligase activity [29, 121]. However, SOCS4 binds with high affinity to the same EGFR phosphotyrosine residue (Y1092) as STAT3; therefore, it may also inhibit STAT3 activation directly by blocking the later ability to dock to EGFR [29]. SOCS4 also has a low affinity for JAK2 and C-KIT, the biological consequences of which remain to be determined [29]. SOCS5 appears able to regulate both RTK and cytokine receptor signalling. Thus, SOCS5 has been shown to negatively regulate EGFR* in vitro* [119, 121] and more weakly IL-6R, LIFR [96], and IL-4R signalling [122]. In fact, by regulating IL-4 receptor signalling, SOCS5 inhibits STAT6 activation and may play a role in T helper (Th)1/Th2 cell differentiation [122]. The principle mechanism of action of SOCS5 in regulating signalling is thought to be targeting proteins for proteasomal degradation, as with EGFR where both its SH2 domain and SOCS box are required for this process [119, 121]. Interestingly, the SOCS5 protein has been found to associate with EGFR independent of ligand stimulation, binding via its N-terminal domain [119].

#### 6.4.2. *In Vitro* Functions of SOCS6 and SOCS7

Like other SOCS proteins, SOCS6 likely primary regulatory role is through ubiquitination and degradation of target proteins [129], using specific interaction with an alternate E3 ligase component named heme-oxidised IRP2 ubiquitin ligase-1 (HOIL-1) [126], and as SOCS2, SOCS6 also has the ability to degrade other SOCS proteins, including SOCS7 [163]. Another aspect of SOCS6 function is its nuclear localisation as its N-terminal domain has been shown to drive SOCS6 localisation to the nucleus, where it appears to negatively regulate STAT3, although the exact mechanism by which SOCS6 regulates STAT3 has not been identified [164]. SOCS6 was also shown to bind to and inhibit the kinase domain of active p56 LCK downstream of TCR, leading to subverted T cell activation* in vitro,* and this effect is thought to be achieved by targeting p56 LCK for ubiquitination and subsequent degradation, with SOCS6 overexpression resulting in inhibition of TCR-dependent IL-2 promoter activity [127]. Following stimulation by SCF, SOCS6 also binds to the juxtamembrane region of C-KIT, thereby regulating activation of members of the MAPK pathway, such as ERK1/2 and p38 [126]. SOCS6 can also bind to FLT3 and negatively regulate its signalling, reducing downstream ERK1/2 signalling and cell proliferation [125]. SOCS6 expression was also found to be induced by IGF-I and reduced by JAK-STAT pathway inhibitors [123]. Perhaps the most known role for SOCS6 has been identified in glucose haemostasis. It is also known that SOCS6 and SOCS7 share greater sequence identity with each other than with other members of the SOCS family (56% within the SH2 domains) and that their expression appears to be coregulated in response to insulin signalling. This insulin role is also supported by SOCS6/7 interaction with the insulin receptor, PI3 K p85 subunit, and IRS2/4 proteins [124, 129].

SOCS6 has been shown to inhibit pathways downstream of the insulin and IGF-I receptors [124]. This was facilitated by direct binding of SOCS6 to the IRS-4 adaptor protein following its phosphorylation in response to IGF-I or insulin and more weakly to IRS-2 in response to IGF-I, allowing it to indirectly associate with the p85 regulatory subunit of PI3 K in response to IGF-I or insulin stimulation [129, 165], thus preventing recruitment of other downstream signalling proteins [129]. SOCS6 may also interact with PIM3, a protein upregulated in *β*-cells in response to glucose stimulation as PIM3 knockout mice showed greatly reduced levels SOCS6 expression in their pancreatic islets, while overexpression of SOCS6 inhibited glucose-induced ERK1/2 activation, suggesting a role for SOCS6 and PIM3 in the negative regulation of ERK1/2 in response to glucose stimulation [166].

SOCS7 was first identified through its ability to interact with the SH3 domain of the adaptor protein Nck and is unique in its possession of a proline-rich N-terminal domain and nuclear localisation motif [27]. Nck is a cytoplasmic receptor tyrosine kinase adaptor molecule [167, 168] which is involved in IGF-IR signalling through its IRS-1 and IRS-2 adaptors [169, 170] and through RAS [171], as well as through SOS-another adaptor in the IGF-IR/RAS/RAF/ERK signalling [172], possibly through its SH3 domain [169]. SOCS7-Nck interaction is now well documented [27, 131, 171], and SOCS7 can act as Nck shuttling protein during its nuclear translocation. Nck nuclear accumulation can occur in response to DNA damage (e.g., UV induced DNA damage), leading to cell cycle arrest and initiation of the p53 apoptotic pathway [131], representing a unique proapoptotic function of SOCS7. SOCS7 is known to be involved in IGF-I signalling control by several other mechanisms. It mediates the proteasomal degradation of IRS-1 docking on the cytoplasmic domain of activated IGF-IR, through SOCS7-SOCS box interactions [130, 139, 173, 174]. It can also interact with and inhibit the function of IRS-2/4 by binding to its SH2 domain [129]. IRS-1 and IRS-2 initiate two signalling pathways downstream of activated IGF-IR, the PI3 K-AKT and the RAS-RAF-MEK/ERK pathways, both necessary in cellular proliferation and differentiation, while IRS-4 serves as docking site for cytoplasmic PLC*γ*-1 leading to its activation and subsequent PKC/ERK activation [175]. SOCS7 can also directly interact with p85, the regulatory subunit of PI3 K-AKT pathway activated downstream of IGF-IR [129, 131]. Furthermore, there is also evidence that SOCS7 may* directly* interact with PLC*γ*-1, similar to its interactions with Nck [27].

Growth factor receptor-bound protein 2 (Grb2: also known as Ash) is another adaptor protein activated during the IGF-IR signalling by its interaction with the receptor bound IRS-1 and IRS-2, leading to the activation of downstream RAS/RAF/ERK pathway involved in the cellular proliferation and differentiation [176]. SOCS7 can interact with Grb2 at this level [27, 130, 171]. All the above SOCS7 interactions with IRS-1, IRS-2, IRS-4, Grb2, and p85 subunit of PI3 K, as well as its SH2 domain interactions with the insulin and EGF receptors are known to target these proteins for proteasomal degradation by recruiting the E3 ubiquitin ligase system [27, 130]. JAK-STAT regulation by SOCS7 is also possible, as SOCS7 inhibits JAK2-STAT3 [27, 130, 171, 177], interacts with STAT5* in vitro* [178], and can alter the nuclear localisation of pSTAT5 [128, 179]. By these interactions with pSTAT3 and pSTAT5, SOCS7 appears to inhibit signalling by leptin and prolactin, respectively [128].

### 6.5. SOCS Proteins Role in the Function of Immune-Regulatory and Proinflammatory IL-2 and IL-12 Cytokine Families

IL-2 and IL-12 constitute important cytokine families that regulate many important cellular functions through STAT activation. Cytokines in the *γ*c family, such as IL-2, IL-7, IL-15, and IL-21, have been shown to play a role in the maturation and function of T cells. These cytokine signals are transmitted mainly by STAT molecules that are regulated directly and indirectly through the activity of SOCS family members [180–182].

The IL-12 family is comprised of IL-12, IL-23, IL-27, and IL-35, and each member interacts with high affinity heterodimeric receptors comprising of the pairing between IL-12R*β*1, IL-12R*β*2, IL-27R*α*, or gp130. The outcome of the response can be proinflammatory or immune suppression. They mediate their biological effects through the activation of STAT pathways hence the importance of SOCS proteins as potential regulatory factors.

For instance, CIS was identified to induce and negatively regulate IL-2 signalling [31, 38, 39] and SOCS1 has been shown to be induced by numerous cytokines* in vitro* and ex vivo, including IL-2 [56]. SOCS1 has also been found to regulate signalling by many receptors* in vitro*, including those for the cytokines IL-2 [24, 56], IL-12 [72].

IFN*γ*/SOCS1 double KO mice developed additional phenotypes, including polycystic kidneys, chronic infections, and inflammatory lesions, which resulted in survival to only 6 months of age [183]. T cell development was also perturbed, including reduced T cells numbers [184], disrupted Th2 responses [185], and a reduced CD4/CD8 ratio [184], as well as abnormal development of Th17 cells [186], resulting from hypersensitivity to cytokines acting via the *γ*c receptor: IL-2, IL-4, IL-7, IL-15 [187], and IL-12 [72].

Like CIS, SOCS2 is induced and regulated signalling by cytokines that activate STAT5, including IL-2 [78, 79] and shown to exert an antagonistic role in the SOCS1- and SOCS3-mediated negative regulation of IL-2 and IL-3 signalling, respectively [79].

SOCS3 has been demonstrated to be induced and regulates signalling by cytokines such as IL-2 [85], and its ability to skew T cell differentiation to the T helper 2 (Th2) phenotype may be due to its competition for the STAT4-binding site (Y800) on the IL-12R*β*2 chain, thus inhibiting IL-12/STAT4-driven polarisation to the alternative T helper 1 (Th1) phenotype [162, 188] or alternatively via its inhibition of IFN-induced STAT1 activation that is also associated with Th1 polarisation [87].

There is a limited research into the role of the rest of the SOCS members in IL-2 and IL-12 signalling. However, SOCS5 transgenic mice were found to have increased peritoneal IL-2 and IFN-*γ*, cytokines involved in the promotion of Th1 differentiation [189], and SOCS6 was shown to bind to the kinase domain of active p56^*lck*^, targeting it for ubiquitination and subsequent degradation, with SOCS6 overexpression resulting in repression of TCR-dependent IL-2 promoter activity [127].

### 6.6. *In Vivo* Functions of SOCS Family

*In vivo* functional redundancy may not only explain the obvious lack of effect in CIS, SOCS5, and SOCS6-deficient mice but also the apparent absence of roles for SOCS proteins in regulation of JAK-STAT-dependent cytokines such as EPO and TPO. It is possible that other SOCS family members can compensate for the loss of individual SOCS proteins, a proposition that remains to be formally tested by the generation of mice with compound SOCS deficiencies. Deletion of the SOCS7 gene had a more dramatic effect, resulting in premature death due to hydrocephalus in C57BL/6 mice, with no obvious defects in glucose homeostasis. Conversely, SOCS7-deficient 129/SvJ mice survived and enhanced insulin signalling was associated with improved glucose tolerance [130, 190]. This example highlights the impact strain background can have on the manifestation of knockout phenotypes.

Table 3 summarises the resulting disorders during SOCS transgenic and knockout* in vivo* experiments.

## 7. SOCS Family and Human Malignancies

Several observations showed a relationship between dysregulated levels of SOCS proteins and cancer development and treatment results. Development and progression of tumors in various human cancers were correlated with both SOCS inactivation [206–213] and inappropriate upregulation of certain SOCS proteins [214–218]. Increased expression of SOCS2 in malignancies like chronic myeloid leukemia (CML) [219, 220] could contribute to transformation by negative interference with other SOCS molecules that normally would suppress tumor development. Persistent expression of SOCS1 and/or SOCS3 is observed in several haematological malignancies such as cutaneous T cell lymphoma (CTCL), chronic myeloid leukemia (CML), ALK+ anaplastic large cell lymphoma (ALCL), and some acute leukemia. In these circumstances, increased expression occurs with constitutive activation of JAK-STAT pathway [214, 221–224]. Moreover, studies have shown that stimulation of prostate cancer cell lines with IL-6 or androgen caused increased expression of SOCS members, while a downregulation with small interfering RNA caused inhibition of proliferation and increased apoptotic rate [225–227]. One possible explanation is that within the cancer microenvironment, tumour cells are sustained by several cytokines, which constantly activate JAK-STAT and other pathways to support cancer cell growth and survival. Expression of SOCS proteins may be a* consequence* of this, rather than a causing mechanism. In these tumours, failure of other negative regulatory pathways acting upon the JAK-STAT pathway, inappropriate regulation of oncogene expression, or inappropriately enhanced oncogene function such as the TEL-JAK2 fusion protein, may well be present, overwhelming the capacity of SOCS proteins to reduce STAT activation. Under these conditions, the inhibitory action of SOCS proteins may not have a significant impact on cancer cell proliferation and survival, despite their increased expression in the cancer cells. Collective evidence therefore suggests that increased SOCS expression may be a consequent mechanism of, rather than a factor contributing to, the cancer phenotype and malignant disease progression. Their involvement as negative feedback regulators of many of the signalling pathways during the malignant transformation makes them truly regarded as tumour suppressors.

### 7.1. SOCS1 Tumour Suppressor Role

Current research demonstrates a significant role for SOCS1 as a tumour suppressor both in haematological and solid tumours.

#### 7.1.1. SOCS1 and Haematological Malignancies

SOCS1 gene has been found to be frequently mutated in both classical Hodgkin lymphoma [228, 229] and primary mediastinal B-cell lymphoma [230], leading to enhanced signalling by STAT5 [228, 230] and STAT6 [229]. Research has also shown that SOCS1 gene was commonly silenced by hypermethylation (and occasionally mutation) in acute myeloid leukemia (AML) [212, 231] and that its reintroduction had caused growth suppression in affected cells [212]. CML patients also demonstrated SOCS1 gene hypermethylation that reverted to an unmethylated state during remission [232]. Some Philadelphia chromosome (Ph)-negative MPDs exhibit SOCS1 hypermethylation, in association with other mutations, such as the hyperactive JAK2V617F mutation [233]. Alternatively, SOCS1 can be overexpressed in Ph-negative MPDs, probably as a compensatory feedback mechanism [234], and this exact phenomenon of SOCS1 constitutive expression and hypomethylation has also been observed in CML [214, 235]. SOCS1 expression in CML also correlated with a poor response to IFN*α* treatment, likely due to a direct effect on receptor signalling [214].

#### 7.1.2. SOCS1 and Solid Tumours

Hypermethylation and silencing of SOCS1 have been commonly reported in solid tumors, including 61% of cervical cancer samples [236] and 45% of oesophageal squamous cell carcinoma samples [237], as well as occasionally in Barrett's adenocarcinoma [238], with combined hypermethylation/gene loss observed in hepatocellular carcinoma [239]. In addition, SOCS1 promoter CpG islands methylation has been associated with transformation of liver cirrhosis to HCC [240, 241]. Hypermethylation-mediated silencing has also been seen in glioblastoma multiforme, along with enhancement of radio resistance, indicative of a proapoptotic function [242]. Hypermethylation of the SOCS1 gene has also been observed in breast and ovarian cancer, where SOCS1 reintroduction was again able to suppress cell growth [243]. In gastric cancer, loss of SOCS1 may be involved in lymph node metastasis and tumour progression [244], and in half of the hepatocellular carcinomas analysed by Nagai et al. [245], its expression is reduced, while restoration of its expression suppressed development and progression of hepatocellular carcinoma cells [246]. Spontaneous colorectal cancer was also seen in SOCS1 knockout mice in an IFN*γ*-dependent manner [247]. Perhaps, this tumour suppressive role for SOCS1 could be attributed—at least in part—to its nuclear localisation and its interaction with p65 and p53 as has been recently suggested [140]. SOCS1 mutation studies indicate that its SH2 domain and SOCS box mediate its binding and subsequent ubiquitin degradation of p65 [140]. In addition, SOCS1 can form complexes with ATM and ATR in the nucleus, contributing to p53 phosphorylation and activation, and promoting p53 mediated senescence in response to oncogenic stimuli [248–250]. This mechanism may explain the spontaneous occurrence of colorectal cancer in SOCS1 knockout mice [247]. Finally, SOCS1 has also been shown to suppress oncogenic forms of VAV [138], C-MET [251], ABL, and C-KIT [248], as well as TEL-JAK2 and BCR-ABL fusions [248].

### 7.2. SOCS2 Tumour Suppressor Role

SOCS2 has been implicated in tumorigenesis, where it has two distinct roles. As has been mentioned above, increased SOCS2 expression in malignancies like CML [219, 220] could contribute to oncogenesis by negative control of other SOCSs functions that normally would suppress tumor development. A similar example exists in solid tumours, as patients with active acromegaly and colonic polyps have shown a significantly increased SOCS2 expression, which mediated a reduction in SOCS1 expression, leading to elevated STAT5b levels, and likely leading to exaggerated GH-mediated proliferation of colonic epithelial cells [252]. In contrast, SOCS2 expression was shown to have a favourable prognostic value in breast cancer [217], and hypermethylation of SOCS2 was detected in ovarian but not breast cancer [243].

### 7.3. SOCS3 Tumour Suppressor Role

Studies have shown that hyperactivation of STAT3 can contribute to tumorigenesis by inducing multiple tumour-promoting genes [253]. Furthermore, reduced expression of SOCS3 has been observed in various human cancers and is associated with constitutive STAT3 activation [253]. For instance, the levels of SOCS3 were found to be inversely correlated with STAT3 activation in regions of human livers with HCC [254]. SOCS3 may also be involved in the suppression of tumour growth and metastasis of several malignancies including malignant melanoma, lung cancer, hepatocellular cancer, and head and neck squamous cell carcinoma [60, 77, 216]. In the case of HNSCC, high rates of SOCS3 methylation correlated with higher grades of dysplasia [255]. Interestingly, SOCS3 represents a good example that SOCS family tumour suppressor activity may not be solely due to their negative feedback role in the JAK-STAT signalling (the other examples are SOCS6 and SOCS7; see below). SOCS3 interferes with the FGF-2 signalling pathway by modulating p44 and p42 phosphorylation in prostate cancer cells. Decreased SOCS3 protein expression results in increased MAPK phosphorylation, whereas SOCS3 overexpression leads to a decreased cellular proliferation and migration [256]. Furthermore, SOCS3 was found to inhibit the proliferation of mesothelioma cells via multiple signalling pathways including JAK-STAT3, ERK, FAK, and p53 pathways [257].

### 7.4. SOCS4 Tumour Suppressor Role

Several studies have suggested a tumor suppressor role for SOCS4. In breast cancer, we reported an inverse relationship between SOCS4 expression and tumor TNM stage and that higher SOCS4 expression might be a predictor of better overall survival [258]. In aggressive hepatocellular carcinoma, an inverse relationship between EGFR expression and SOCS4 and SOCS5 expression has also been reported [259]. SOCS4 expression was also found to be significantly lower in gastric cancer compared to noncancerous gastric tissue, along with hypermethylation of CpG sites in the promoter region of the SOCS4 gene leading to its silencing [260].* In vivo* studies using mouse models also suggest a tumor suppressor role for SOCS4 in epithelial cells via RUNX1-mediated repression of the SOCS4 promoter, leading to decreased SOCS4 levels and increased STAT3 activity, promoting tumor development [261].

### 7.5. SOCS5 Tumour Suppressor Role

Tumour suppressor activity was also identified in SOCS5. In breast cancer tissue, SOCS5 expression was inversely related to the tumour TNM stage [258], and, in a recent report, exogenous expression of SOCS5 (as well as SOCS1 and SOCS3) in the highly aggressive anaplastic thyroid cancer cells has been shown to reduce or abolish STAT3 and STAT6 phosphorylation and PI3 K/AKT pathway activation and resulted in alteration in the balance of proapoptotic and antiapoptotic molecules and sensitisation to chemotherapeutic drugs* in vitro* [262]. Likewise, exogenous expression of SOCS3 was found to significantly reduce tumour growth and potently enhance the efficacy of chemotherapy* in vivo* [262].

### 7.6. SOCS6 Tumour Suppressor Role

More recently, tumour suppressor activity was also identified in SOCS6. SOCS6 is downregulated in a variety of cancers and has capacity to inhibit tumorigenesis when expressed in cell lines derived from gastric cancer (AGS and AZ-521) as well as nonsmall cell lung cancer (H1299) and kidney (HEK293) [263]. It is also downregulated in recurrent primary lung squamous cell carcinoma [264], as well as in cancers of the liver and the thyroid gland [265].

Loss of tight regulation of the stromal cell factor (SCF) receptor, C-KIT, can lead to the development of several human cancers [266, 267], and SOCS6 can bind directly to the juxtamembrane (JM) region of C-KIT following SCF stimulation and phosphorylation of murine C-KIT Y567 (human Y568) [126]. Overexpression of SOCS6 in a Ba/F3-KIT cell line caused a 40% decrease in SCF-dependent cell proliferation and a similar reduction in signalling through ERK1, ERK2, and p38. [126]. SOCS6 SH2 domain is essential for the interaction with C-KIT, while the SOCS box interaction with Elongin B/C contributes to SOCS6 stability. Moreover, SOCS6 is an E3 ubiquitin ligase for C-KIT* in vitro* and modulates its stability* in vivo* [268].

### 7.7. SOCS7 and CIS Tumour Suppressor Role

Less data is available on the tumour suppressor activity of SOCS7 and CIS. In a recent study, prostate cancer LNCaP-S17 cells were found to be resistant to exogenous IL-6-induced neuroendocrine differentiation and hence were less aggressive due to increased levels of CIS and SOCS7 that block activation of JAK2-STAT3 pathways [177]. Furthermore, in colonic cancer cell line HT-29 that constitutively expresses STAT6, there is downregulation of CIS and SOCS7 (in addition to SOCS1, SOCS3, and SHP1) [269]. Further data reported by our group demonstrated a favourable role for SOCS7 in breast cancer. An inverse relationship between SOCS7 mRNA expression and the TNM stage as well as the tumour grade of breast cancer was found. Furthermore, higher SOCS7 expression may be a predictor of better disease-free survival and overall survival in breast cancer [258]. More data demonstrated an involvement of the SOCS7 in the negative control of IGF-I/PLC*γ*-1 signalling in MCF7 and MDA-MB-231 breast cancer cell lines, which consequently limit their growth and migrational functions [270].

## 8. SOCS and Inflammation-Associated Cancer

Abundant evidence now exists that SOCSs are key negative regulators of the inflammatory response and are essential in maintaining normal cellular homoeostasis. This would be in line with the tumour suppressive ability of SOCS family as* >*20% of all malignancies are initiated or exacerbated by inflammation.

For instance, most human hepatocellular carcinomas (HCCs) result from hepatitis C virus (HCV) infection [271, 272]. The expression of SOCS1 gene is often silenced in these tumours by hypermethylation of CpG islands of the SOCS1 promoter [241]. SOCS1 is one of the most frequently methylated genes (65%) in HCCs, and the deletion of SOCS1 in tumour cells might enhance IL-6-mediated cell proliferation. Supporting this is the finding that SOCS1^+/−^ mice are consistently shown to be hypersensitive to dimethylnitrosamine-induced hepatocarcinogenesis [241].

The full picture, however, may not be that simple. It has been found that silencing of SOCS1 was frequently observed even in premalignant HCV-infected patients [241]. Liver injury is associated with STAT1 hyperactivation and reduced STAT3 activation [254, 273]. Therefore, reduced expression of SOCS1 might enhance tissue injury and inflammation by hyperactivation of STAT1, promoting the turnover of epithelial cells and enhancing their susceptibility to oncogenesis.

The importance of SOCS1 for inhibition of inflammation-associated tumour development is supported by the finding that a strain of SOCS1^−/−^ mice, in which SOCS1 expression is deleted in all types of cells except T and B cells, developed chronic colitis and colon tumours [247]. This strongly suggests that chronic activation of the IFN*γ*-STAT1 pathway that occurs in the absence of SOCS1 causes colitis-induced colon tumours. Therefore, SOCS1 is a unique antioncogene that prevents carcinogenesis by suppressing chronic inflammation.

More recent data suggest that administering probiotics can reduce H pylori induced gastritis and therefore the risk of associated gastric cancer by the increased cellular expression of SOCS2 and SOCS3 [274].

A recent model of inflammation-associated tumorigenesis was proposed by Yoshimura in 2009 [275]. In this model, initiation occurs with mutation in one of the molecules regulating the pathways controlling cell division, survival, and senescence. Persistent inflammation leads to tissue damage and increased cellular turnover. Nitric oxide (NO) and reactive oxygen species (ROS) from inflammatory cells may induce DNA damage, which leads to the emergence of cells with a high risk of malignant transformation. STAT1 plays a positive role in nontumour inflammatory regions in this early stage, and SOCS1 silencing in pretumour cells results in strong and persistent STAT1 activation, which induces apoptosis and tissue damage, leading to further DNA damage and cell regeneration which may promote the emergence of malignant cells. Then, promotion occurs by cellular and extracellular signals activated by cytokines from inflammatory cells or stromal cells, leading to immortalized cells that are resistant to growth-inhibitory signals, apoptosis, and antitumour immunity.

Reduced SOCS3 expression has also been observed in a variety of inflammation-related human cancers and cancer cell lines and correlated with strong STAT3 activity in these cells [206, 276–280]. Studies showed that, during colitis-associated colonic tumorigenesis, IL-6 in the intestinal lamina propria enhances STAT3-dependent proliferation of tumor-initiating cells and protection of premalignant intestinal epithelial cells from apoptosis [281, 282]. SOCS3 was found to limit inflammation-associated oncogenic transformation in the colon, via regulation of STAT3 and NF*κ*B [283], while in ulcerative colitis, loss of SOCS3 expression was observed in the areas of colonic dysplasia [284]. SOCS3 was protective against hepatitis-induced HCC, with loss of SOCS3 leading to reduced apoptosis and increased proliferation [254]. Constitutive STAT3 activation in tumour cells contributes to an expansion of tumour cells by promoting cell proliferation, survival, angiogenesis, and tissue remodelling. SOCS3 silencing is one of the mechanisms for constitutive STAT3 activation. However, the mechanism of the reduction of SOCS3 expression in tumours has not been established [275].

## 9. Silencing and Dysregulation of SOCS Genes during Tumorigenesis

Tumour suppressor genes prevent the formation of tumour cells by enforcing anticancer mechanisms such as cell growth arrest, DNA repair, and apoptosis. The loss of function of a tumour suppressor gene can increase the probability of the formation of a tumour. Common examples are p53 and pRb (retinoblastoma family protein) which are major tumour suppressor proteins. p53 is involved in apoptosis and cell cycle regulation and is one of the most mutated genes in human cancers with the restoration of p53 function leading to the regression of tumours [285]. pRb prevents the replication of damaged DNA and is dysfunctional in many cancers [286]. The loss of pRb function leads to the overexpression of the mitotic checkpoint protein, the mitotic arrest deficient protein 2 or MAD2, which in turn promotes aneuploidy, a hallmark of many cancers [287]. Dysregulation of the JAK-STAT signalling pathway has been implicated in malignant progression. Many human cancers including hepatocellular carcinoma (HCC), nonsmall-cell lung cancer, mesothelioma, head and neck squamous cell carcinoma (HNSCC), cholangiocarcinoma, Barrett's adenocarcinoma, and myeloproliferative diseases (MPDs) demonstrate constitutive STAT phosphorylation, and this is frequently accompanied by hypermethylation silencing of one or more SOCS genes [206, 208, 212, 238, 255, 279, 288]. SOCS proteins may play an important tumour suppressor role preventing the dysregulation of such pathways. Supporting this is the fact that experimental overexpression of SOCS proteins in cancer cells reduces STAT activity, inhibits proliferation, and induces apoptosis of these cells [206, 208, 240, 255]. Loss of SOCS expression may therefore facilitate tumour progression in conjunction with other oncogenes. However, the process that induces SOCS gene silencing by mechanisms such as methylation is not fully clear.

### 9.1. Mutations and Deletions

Generally, point mutations, deletions, rearrangements, and duplications in tumour suppressor genes are frequently involved in malignant cell transformation. For instance, mutational inactivation of both Rb1 alleles is the primary molecular cause of retinoblastoma. Approximately 10% of retinoblastomas are inherited and are caused by germ-line transmission of one mutated Rb1 allele and loss of the remaining wild-type allele in somatic retinal cells [289]. Similarly, at least 29 different breast cancer susceptibility gene 1 ( BRCA1) germ-line mutations have been linked to women's breast and ovarian cancers [290].

In relation to SOCS, a biallelic mutation in SOCS1, resulting in a defective SOCS1 SOCS-box, was observed in the primary mediastinal large B-cell lymphoma cell line, MedB-1 [291]. Similarly, in the primary Karpas 1106P lymphoma cell line, a large biallelic chromosome deletion on 16p13.13 which includes SOCS1 was observed, again resulting in constitutive JAK-STAT signalling [270].

Additionally, SOCS1 and SOCS3 epigenetic silencing were occasionally detected, and SOCS1 was frequently mutated in diffuse large B-cell lymphoma and polymorphic posttransplant lymphoproliferative disorders, possibly as a cause of aberrant somatic hypermutation [292].

SOCS1 mutations were also present in 8/19 laser-microdissected Hodgkin and Reed-Sternberg cells of classical Hodgkin lymphomas, which correlated with nuclear accumulation of pSTAT5 [228]. As in other SOCS members, the SOCS box enables SOCS1 to form a multisubunit E3 ligase complex to target SOCS1-associated proteins to the ubiquitin-proteasome pathway [135]. It is thought that impaired SOCS1-mediated JAK2 degradation results in sustained JAK2 activation and low turnover of JAK2 protein leading to lymphomas [228].

### 9.2. Methylation

DNA methylation involves the addition of a methyl group at position C-5 of the cytidine ring in the context of a CpG dinucleotide, often in gene promoter regions, leading to transcriptional silencing of that gene. Aberrant methylation is the best-studied epigenetic abnormality in tumorigenesis, and hypermethylation of tumour suppressor gene promoters including APC (adenomatous polyposis coli), p16, BRCA1, Rb, and MDM2 (murine double minute 2) is often associated with cancer development [293]. Similarly, IL-6, a proinflammatory cytokine, enhances and maintains hypermethylation of the p53 tumour suppressor gene and the hHR23B gene, a key component of the nucleotide excision repair promoter in the multiple myeloma cell line KAS-6/1 [294]. Conversely, IL-6 induced hypomethylation of EGF receptor, leading to its enhanced expression and growth of cholangiocarcinoma cells [295]. These data suggest that DNA hypo- and hypermethylation are important mechanisms that could contribute to inflammation-associated tumorigenesis. Since SOCS proteins have recently been added to this list of tumour suppressors, a great deal of interest has focused on the methylation status of SOCS in human tumours. Aberrant methylation of SOCS1 CpG islands has been reported in lymph node metastasis, advanced human gastric carcinoma [244, 296], oesophageal carcinoma [237], hepatocellular carcinoma [297], myeloma [298, 299], pancreatic carcinoma [300], cervical carcinoma [236], and breast carcinoma [243] where SOCS1 silencing in tumour cells is believed to enhance IL-6-mediated cell proliferation. In HCCs, approximately 65% of primary tumours showed abnormal SOCS1 methylation in exon 2 and restoration of SOCS1 expression suppressed cell growth and resulted in apoptosis [244], and the methylation frequency of SOCS1 gene was 82.6% in a cohort of 115 human HCC samples [301]. Likewise, the loss of SOCS3 expression is thought to confer a cell growth advantage and promote cell migration due to enhanced JAK-STAT and FAK signalling, respectively. SOCS3 is methylated in approximately 90% of head and neck squamous cell carcinoma samples, 74% of oesophageal Barrett's adenocarcinomas, 60% of melanomas, in HCC, and in nonsmall cell lung carcinomas [206, 238, 255, 276, 277]. It is also methylated and transcriptionally silenced in nearly 60% of AMLs and 40% of chronic myeloproliferative disorders [302]. Similar epigenetic silencing of SOCS3 has been seen in cholangiocarcinoma and colonic cancer, resulting in enhanced IL-6/STAT3 signalling and reduced apoptosis [279, 303]. SOCS3 hypermethylation was also seen in glioma [304] and also in prostatic cancer where aberrant methylation of SOCS3 was found in 39% of cases of prostate cancer in contrast to all benign (BPH) cases and normal control which showed a SOCS3 promoter nonmethylation [305]. Further demonstration of SOCS silencing in tumorigenesis is seen in colon cancer cell lines and was linked to constitutive STAT expression. Colonic cancer HT-29 cells with high STAT6 expression phenotype (STAT6^high^) exhibited low constitutive expression of STAT6-negative regulators SOCS1 and SHP1 because of gene hypermethylation, with the opposite findings in STAT6^null^ cells [306, 307]. Similar to SOCS1 and SHP1, STAT6^high^ HT-29 cells expressed low constitutive mRNA of SOCS3 and SOCS7 than STAT6^null^ colonic cancer Caco-2 cells [269].

Other examples include SOCS6, as its loss was reported in more than 50% of patients with gastric or colorectal cancer, with SOCS6 inactivation predominantly caused by allelic loss or promoter hypermethylation [263, 308]. However, in the case of colorectal cancer, this did not correlate with disease-free survival or overall survival [309].

### 9.3. Aberrant SOCS Phosphorylation and the Role of PIM Kinases

Oncogenic kinases may use posttranslational modification of SOCS proteins, such as aberrant tyrosine phosphorylation, to prevent the negative regulation of pathways required for cell growth and proliferation For instance, recent evidence suggested that BCR-ABL-dependent tyrosine phosphorylation of SOCS1 and SOCS3 occurs mainly on Y155 and Y204 residues of SOCS1 and on Y221 residue of SOCS3, leading to their binding to BCR-ABL oncogenic protein and loss of their inhibitory effect on the activation of JAK-STAT signalling [310].

As previously stated, SOCSs can act as E3 ubiquitin ligases to accelerate the ubiquitination and degradation of SOCS binding partners [35]. Posttranslational modification of SOCS proteins may be a mechanism utilized by oncoproteins to circumvent their degradation by E3 ubiquitin ligases. An example of this mechanism is SOCS1 phosphorylation. Serine phosphorylation can regulate the stability of SOCS1 and its capacity to interact with Elongin C. For instance, v-Abl-mediated phosphorylation of SOCS1 disrupts its binding to the Elongin B/C complex, thus blocking the SOCS1-mediated JAK degradation contributing to the transformational properties of v-Abl [311, 312]. Knowing that increased expression of PIM kinases—a group of serine/threonine kinases—has been associated with several cancers including lymphomas, prostate cancer, and oral cancer [313, 314], it is now believed that this association is linked to their induction by v-Abl.

v-Abl induces several serine/threonine kinases, which could be responsible for the v-Abl-mediated phosphorylation of SOCS1. These include the PIM kinase family: PIM1, PIM2, and PIM3 [315, 316]. Mice deficient in all three family members have reduced body size and impaired proliferation of haematopoietic cells in response to growth factors [317].

The phenomenon of phosphorylation preventing the formation of an E3 ubiquitin ligase is not unique to SOCS1. Tyrosine phosphorylation of SOCS3 is important in regulating its stability. Phosphorylation of SOCS3 can occur at two tyrosine residues in the SOCS box, Y204 and Y221, resulting in the inhibition of the SOCS3-Elongin C interaction and SOCS3-mediated degradation. Furthermore, when both tyrosines were mutated to phenylalanine, this had delayed the turnover of SOCS3 and increased its half-life [99, 318].

### 9.4. Other Silencing Ways of SOCSs: The Role of LCK Kinase

Aberrant expression or activation of LCK kinase, an SRC protein tyrosine kinase, has been reported in both lymphoid and nonlymphoid malignancies, predominantly through activation of STAT5b [319]. SOCS1 and SOCS3 are not expressed in LCK-transformed leukemias. This is thought to be either due to hypermethylation (e.g., SOCS1) or due to unrelated mechanism (e.g., SOCS3) [320]. Furthermore, exogenous expression of SOCS1 or SOCS3 leads to reduced cell proliferation and increased apoptosis in LCK-transformed cells, which is thought to be due to the attenuation of LCK kinase activity [320]. Downstream STAT5 activity is also inhibited as shown by reduced STAT5 tyrosine phosphorylation and* in vitro* DNA binding [320].

## 10. Concluding Remarks

Over the past decade, following the discovery of the SOCS protein family, we have extended our understanding of the structure and function of these proteins. SOCS proteins act as simple negative feedback regulators, and they also play a part in the fine tuning of many cellular functions such as those involved in the immune response and inflammation, but more recently, there has been a growing evidence of their tumour suppressor role.

Further research should be carried out to shed more light on their role in downstream signalling regulation during the cellular transformation and proliferation in the early stages of human cancer development. Our understanding of these mechanisms may identify new therapeutic applications.

## Figures and Tables

**Table 1:** Cytokine activation of janus kinases (JAKs).

Cytokine receptor	Associated JAK(s)
Interferons	
IFN*α*/*β*	JAK1 [8] and JAK2 [9]
IFN*γ*	JAK1 [9, 10] and JAK2 [9, 10]
IL-10	JAK1 [11] and TYK2 [11]
IL-6	JAK1 (IL-6R *α* chain and gp130) [12]
Shared *γ*_c_ receptor (IL-2, IL-4, IL-7, IL-9, and IL-15)	JAK1 [13] and JAK3 [13]
Shared *β*_c_ receptor (IL-3, IL-5, and GM-CSF)	JAK2 [14]
Homodimer receptors (GH, EPO, prolactin, and TPO)	JAK2 [15]

Abbreviations: GM-CSF: granulocyte monocyte colony stimulating factor; EPO: erythropoietin; TPO: thrombopoietin.

**Table 2:** SOCS family members associate with a variety of signalling proteins and inhibit signalling by many cytokines.

Name	Induced and/or Inhibits signalling by	Associates with
CIS	IL-2 [37, 38]; IL-3 [31, 39]; IL-6 [23]; IL-9 [40]; IL-10 [41]; PRL [42–44]; EPO [39]; IGF-I [45]; GH [37, 46, 47]; TPO [23, 48]; G-CSF [49]; GM-CSF [31, 39]; IFN*α* [50]; IFN*γ* [23]; TNF*α* [23]; Leptin [51]; TSLP [52]; CNTF [53]	IL-2 R [38]; IL-3 R [31]; EPO R [31]; GH R [47], [54]; PCK*θ* [55]; TCR [55]
SOCS1	IL-2 [24, 56]; IL-3 [24]; IL-4 [32, 57, 58]; IL-6 [23, 32]; IL-13 [59]; GH [47, 54, 60]; PRL [42]; EPO [23, 24]; LIF [32]; IFN*γ* [23, 61]; IFN*α*/*β* [61, 62]; OSM [23]; TSLP [52]; TPO [23]; IGF-I [45]; G-CSF [32]; CNTF [53]; TNF*α* [63]; LPS [64, 65]; INS [66]; TSH [67]; CpG DNA [68]	JAK1 [24]; JAK2 [24, 28]; JAK3 [24]; GRB2 [69]; VAV [69]; FGF R [33]; PYK2 [33]; GH R [47, 54, 60]; KIT R [33, 69]; FLT3 R [69]; IGF-I R [45, 70], INF*α* R [61]; INF*γ* R [61]; EPO R [24]; PRL R [42, 44], LIF R [32]; TNF*α* R [63]; IL-2 R [24, 56]; IL-3 R [24]; IL-4 R [57]; IL-6 R [32]; Il-7 R [71]; IL-12 R [72]; Il-15 R [73]; TPO R [23]; TSLP R [52]; OSM [23]; Leptin R [74]; TLR [75]; INS R [76]
SOCS2	IL-1*β* [77]; IL-2 [78, 79]; IL-3 [23, 79]; IL-4 [23]; IL-6 [77, 80]; IL-15 [81]; GH [47, 60]; PRL [42]; LIF [23, 78]; IGF-I [45, 70]; EPO [23, 82]; EGF [83]; GM-CSF [23]; G-CSF [23]; IFN*α* [50]; IFN*γ* [23, 77]; CNTF [53]; INS [66]	IGF-I R [70]; PRL R [42]; GH R [47, 54]
SOCS3	IL-1*β* [84]; IL-2 [78, 85]; IL-3 [78, 86]; IL-4 [57]; IL-6 [87–89]; IL-9 [40]; IL-10 [41]; IL-11 [90]; IL-13 [23]; IL-22 [91]; GH [23, 47, 54, 60, 92]; PRL [42]; EPO [93, 94]; TPO [95]; LIF [60, 96]; IFN*α* [61]; IFN*γ* [61]; G-CSF [23]; GM-CSF [97]; TNF*α* [98]; IGF-I [45, 66]; EGF [99, 100]; PDGF [99]; BFGF [101]; TSH [67]; CNTF [53]; Leptin [51, 102–104]; OSM [61, 105]; INS [66, 104, 106]; CT1 [107]	IL-1*β* R [108]; IL-2 R [85]; IL-4 R [57]; IL-6 R [96]; IL-9 R [40]; IL-11 R [90]; IL-23 R [109]; IL-27 R [110]; PRL R [42, 44]; LIF R [60]; IFN*α*/*β* R [61]; IFN*γ* R [61]; G-CSF R [111]; LCK [33]; FGF *R* [33]; PYK2 [33]; GH R [47, 54, 60]; EPO R [93]; Leptin R [102, 112]; gp130 [93, 113–115]; IGF-I R [45, 70, 116]; CNTF R [53]; OSM R [105]; INS R [104]; CT1 R [107]; CD28 [117]; Calcineurin [118]
SOCS4	EGF [119]; LIF [120]	EGF R [29, 119, 121]; JAK2 [29]; C-KIT [29]
SOCS5	IL-6 [96]; EGF [119]	IL-4 R [122]; IL-6 R [96]; EGF R [119, 121]; LIF R [96]
SOCS6	IGF-I [123]; INS [124]; FLT3 [125]; SCF [126]	IGF-I R [123]; INS R [124]; FLT3 R [125]; SCF R [126]; TCR [127]
SOCS7	GH [77, 128]; PRL [77, 128]; IGF-I [129]; INS [129]; EGF [27]; Leptin [128]	GH R [128]; PRL R [128]; INS R [129, 130]; IRS-1 [130]; IRS-2 [129]; IRS-4 [129]; PI3K (p85) [129]; Grb2 [27]; EGF R [27]; Leptin R [128]; Ash [27]; Nck [27, 131]; PLC*γ* [27]

Abbreviations: R: receptor; PKC: protein kinase C; OSM: oncostatin M; Tpo: thrombopoietin; BFGF: basic fibroblast growth factor; CNTF: ciliary neurotrophic factor; CT1: cardiotrophin-1; TSLP: thymic stromal lymphopoietin; TCR: T cell receptor; INS: insulin; SCF: stem cell factor.

**Table 3:** *In vivo* disorders resulting from SOCS proteins manipulation.

Gene	Knockout phenotype	Transgenic phenotype	Main affected cytokines	Reference
CIS	(?) Increased haematopoiesis, disturbed lactation, and increased susceptibility to infections with single nucleotide polymorphism (SNP) at CIS promoter position 292	Reduced weight, defective mammary gland development, altered T and NK cell responses	STAT5 signalling (GH, EPO, IL-2, IL-3, and PRL)	[37, 93, 94, 191]

SOCS1	Multiorgan inflammation, neonatal lethality, lymphocyte apoptosis, and haematopoietic infiltrations	Disturbed T-lymphocyte development and spontaneous T cell activation	IFN*γ*, IFN*α*, IL-4, and IL-12	[57, 192–195]

SOCS2	Gigantism	Gigantism	GH and IGF-1	[79, 149, 163, 196–198]

SOCS3	Embryonic lethality, placenta defects, disturbed erythropoiesis, and enhanced response to G-CSF	Embryonic lethality, increased Th2 differentiation, and reduced pancreatic *β* cell proliferation	gp130, IL-2, IL-6, G-CSF, leptin, and EPO	[94, 111, 199–203]

SOCS4	?	?	?	

SOCS5	(?) No obvious phenotype (redundancy with SOCS4?)	Disturbed Th2 differentiation, increased peritoneal IL-2 and IFN*γ*, and decreased lethality from peritonitis	IL-4 and EGF	[129, 189, 204, 205]

SOCS6	Mild growth retardation (redundancy with SOCS7?)	Improved glucose and insulin tolerance	Insulin (?)	[129, 165, 204]

SOCS7	Hydrocephalus, 50% mortality, Hyperinsulinemia	?	Insulin	[130, 190]
